# Electrophysiological fingerprints of healthy cervical epithelial and HeLa cells: Membrane potential, zeta potential and passive electrical properties

**DOI:** 10.1371/journal.pone.0337538

**Published:** 2025-12-17

**Authors:** Rashedul Hoque, Srdjan Cirovic, Michael Pycraft Hughes, Fatima H. Labeed

**Affiliations:** 1 Centre for Biomedical Engineering, School of Mechanical Engineering Sciences, University of Surrey, Guildford, Surrey, United Kingdom; 2 Department of Biomedical Engineering and Biotechnology, Khalifa University of Science and Technology, Abu Dhabi, UAE; 3 Department of Biology, United Arab Emirates University, Al Ain, UAE; CHOC Children's Hospital - UC Irvine, UNITED STATES OF AMERICA

## Abstract

There is a growing appreciation that cellular electrical mechanisms play an important role both in cell regulation, and in cell dysregulation in diseases such as cancer. These electrical mechanisms are measured using several different methods, which yield characteristics including the membrane potential, capacitance and conductance, the extracellular (ζ) potential and cytoplasm conductivity. However, since these are measured using different techniques, the combination of all of these (the *cancer electrome*) has yet to be described. In this paper, we report on the difference between the electromes of cervical cancer cell line HeLa, with clinically-derived primary cervical epithelial cells. These were investigated using dielectrophoresis (DEP) and ζ-potential, with these data then being used to calculate the membrane potential *V_m_.* Results indicate significant differences in membrane conductance and capacitance, membrane potential, ζ-potential and cytoplasm conductivity between the two cell types. Furthermore, treatment with the K^+^ blocker Tetraethylammonium caused distinct alterations in electrophysiology of the two lineages, pointing towards different roles for K^+^ in cancer and normal cells. This work presents a novel and cost-effective approach, combining five distinct electrical properties to form a “fingerprint” to characterize and discriminate healthy and malignant cells in a label-free, rapid manner.

## 1. Introduction

Cervical cancer is the fourth most common cancer worldwide and the fourth leading cause of cancer-related death among women, due largely to persistent human papillomavirus (HPV) infection typically via sexual contact [1,2]. Cervical cancer disproportionately occurs in low and middle income countries (LMICs), which face significant challenges due to scarce resources for disease screening and management [1,3]. Future projections predict that cervical cancer will remain among the most prevalent and costly cancers in LMICs [4]. Cervical cancer management also remains a significant economic burden in developed nations, with the current national cervical screening programme in England costing approximately £175 million per year [5]. The wider socioeconomic burden of cervical cancer in the UK (including healthcare, loss of workplace productivity, losses due to a patient’s inability to undertake caregiving tasks, and quality-of-life losses) was estimated to be £691 million in 2023, with the lifetime socioeconomic burden per person being approximately £210,000 [6]. Despite early diagnosis being associated with improved prognosis, many patients do not take routine cervical screening [6,7]. Lapsed routine screening increases the likelihood of late cervical cancer diagnosis, which in turn increases the requirement for cervical cancer treatment at late stages [8]; late diagnosis is the primary driver for the elevated economic costs associated with cervical cancer [6]. In 2020, the World Health Organisation (WHO) published a global strategy to achieve cervical cancer eradication by the end of the 21^st^ century. Several key targets were recommended by 2030, including the routine screening of 70% of women using “high-performance tests” at the ages of both 35 and 45 [9]. The current standard method of cervical screening is Papanicolaou (Pap) smears investigated by microscopy. However, these have reduced sensitivity at higher grades of cervical intraepithelial neoplasia (CIN), also exhibiting notable inter-operator variability [10,11]. In contrast, HPV testing, while having higher sensitivity to higher CIN grade lesions than Pap smears, has reported lower specificity [12] and carries a potential risk of misclassifying HPV-positive women due to fluctuations of viral load during the menstrual cycle [13]. Both screening methods are also costly, resource and personnel-intensive. Consequently, LMICs have largely settled for visual inspection with acetic acid (VIA), which is cheaper (reported at typically $3.50 - $34 per examination [14,15]) but less accurate [14,15]. To meet the challenges of cervical cancer management WHO goals in resource-scarce LMICs, an alternative, cost-effective “high-performance” screening test is required. To address this, other biomarkers of cancer state are needed.

**Fig 1:**
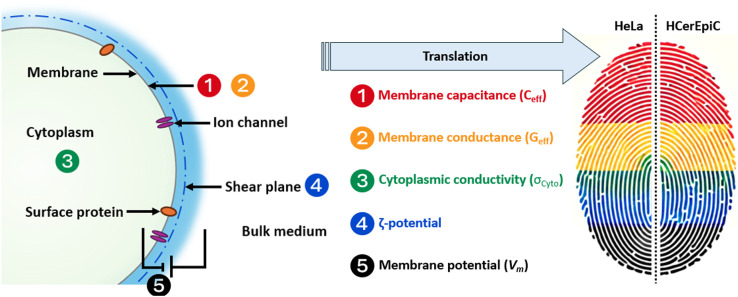
Schematic showing the electrophysiological parameters of a cell, which are altered in malignancy. In the context of cervical cancer, membrane capacitance (1) is elevated; membrane conductance (2) is reduced and there is altered cytoplasmic conductivity (3); the surface charge, ζ-potential (4), is depolarised; membrane potential (5) is also depolarised. Integrating all electrophysiological parameters translates to distinct “fingerprints” to identify cancer from normal cells.

Beyond conventional molecular markers and visual inspection, an alternative approach is to measure the electrical characteristics of cells, commonly grouped under the general term of “cell electrophysiology.” This term is often used to refer to the cell membrane potential (*V*_*m*_), the electrical potential arising between cytoplasm and bulk medium due to partitioning of ions (K^+^, Na^+^ and Cl^-^) across the membrane, giving rise to diffusion potentials [16]. However, it encompasses many other measurands, such as the conductance and capacitance of the cell membrane, the conductivity of the cytoplasm, and the extracellular (ζ) potential, as shown schematically in Fig 1. These are increasingly considered the “cellular electrome” [16]; a set of parameters that are interconnected, though not directly proportional, that collectively point to a role in cell physiology. For example, the ζ-potential is the extracellular potential a short distance (approximately 1 nm) outside the cell surface which arises due to charges on the cell surface, at the boundary of the so-called “stagnant layer” of water that is hydrodynamically bound to the cell surface [17]. It defines how cells regulate electrostatic interactions between that surface and other charges, for example dictating whether two objects will attract (due to van der Waals forces) or repel (due to like-sign electrostatic repulsion). Whilst this was previously assumed to be related only to the surface chemistry of the cell membrane, recent work by Hughes and colleagues [16,17,18] has shown that cells dynamically alter *V*_*m*_ to change ζ-potential, in turn altering how they interact with charged bodies in their environment including ions, molecules and other cells.

The electrome can be measured using a range of techniques. The gold standard technology is “patch clamp”, but this is slow and cumbersome, typically only permitting measurement of a single-digit numbers of cells per day. New approaches are emerging that are faster, cheaper and more accessible. For example, Dielectrophoresis (DEP) has gained particular interest in the field of biomedicine, by examining the motion of cell populations in nonuniform electric fields to determine distinct electrophysiological properties (conductivity and capacitance of membrane and cytoplasm), in a label-free manner [19,20]. It has been used to characterise various cancer cells [21–24], as well as circulating tumour cells [21,25]. Biomedical applications of DEP have included characterising epithelial cancers [26,27], stem cells [28] and measuring drug-dose response [29].

Evidence has emerged in recent years of consistent, systemic changes observed in cells as they transform from healthy to cancerous. This “cancer electrome” has been characterised by depolarised *V*_*m*_ [30,31]. Differences in passive electrical properties measured using DEP have demonstrated the potential for diagnosis, distinguishing between oral cancer and healthy epithelia using brush biopsies [32] and bladder cancer from healthy cells using urine specimens [33], with sensitivity and specificity comparable to molecular adjuncts without the need for long preparation times and markers. Analyses of cancer ζ-potentials have been far more limited [17], with only limited data on breast cancer cells (MCF-7) and healthy breast epithelial cells (MCF-10A) suggesting that ζ-potential also depolarises from −31.2 mV to −20.3 mV [34]. Until now there has not been a comprehensive study of the cancer electrome using a combination of all three approaches; while *V*_*m*_, DEP and ζ-potential analyses have each demonstrated the ability to measure distinct electrophysiological properties of cells, research involving these modalities has been conducted in isolation.

In this paper, we present the first examination of the electromic differences between an epithelial cancer line and normal tissue associated with the same site, using DEP and ζ-potential techniques to comprehensively characterise the electrophysiology of HeLa cells and primary normal cervical cells, and establishing distinct “fingerprints” for rapid, label-free profiling. We use the well-characterised HeLa cervical cancer cell line as our cancer model, and compare this to primary human cervical epithelial cells, which avoids the potential introduction of cancer-like traits associated with the immortalisation of cell lines. Cells were analysed using DEP and ζ-potential analysis across multiple conductivities, with the former being used to derive the mean membrane potential *V*_*m*_, to derive a comprehensive view of the electrome of both cell types. As an additional test, we treated both cell types with the broad-spectrum K+ blocker tetraethylammonium (TEA) which acts by interacting with the K^+^ pore in ion channels [35]. Since a reduction in K^+^ in cancer [30] may be the cause of the depolarisation observed across cancer cells, we sought to investigate how TEA treatment would affect the electrome of both cell types. Results show significant differences in the electrophysiological makeup between healthy and cancerous cells both before and after treatment with TEA, with potential benefits both for cancer biology and cancer diagnosis.

## 2. Materials and methods

### 2.1 HeLa and primary cervical cell culture

All cell culture supplements and chemicals were purchased from Sigma-Aldrich (Gillingham, UK) unless stated otherwise. HeLa human cervical carcinoma cells were obtained from ATCC (Manassas, USA). Once thawed, HeLa cells were cultivated in Dulbecco’s Modified Eagle Medium (DMEM), with 10% heat inactivated foetal bovine serum, 2mM L-glutamine, 1% non-essential amino acids and 1% penicillin-streptomycin, incubated at 37°C, 5% CO_2_. The medium was changed every 48-72h and cells were passaged using trypsin-EDTA when over 70% confluent. HeLa cells were seeded at 1.3x10^4^ cells/mL in T75 flasks. Primary human cervical epithelial cells (HCerEpiC) were obtained from Innoprot (Derio, Spain), where once thawed, were cultured using Innoprot proprietary cervical epithelial medium; this sterile basal medium comprised 1% cervical epithelial growth supplement and 1% penicillin-streptomycin solution. Cells were cultured in collagen coated vented culture flasks (Zenbio, USA) at 37°C, 5% CO_2_, where medium was changed every 48-72h and cells were passaged using low concentration 0.05% trypsin-EDTA when confluent.

### 2.2 Drug treatments

Once around 80% confluent, the harvested HeLa cells and HCerEpiCs were centrifugated at 125 G, resuspended in fresh culture medium and equally split into two tubes. One vial was untreated and represented a standard control. The other vial was treated with 10 mM treatment of the broad-spectrum K^+^ blocker tetraethylammonium chloride (TEA) for 2 hours prior to electrophysiological analysis, an established and well-tolerated procedure used to induce transient membrane depolarisation without cytotoxicity [30,36,37].

### 2.3 DEP and ζ-potential experiments

DEP experimental medium was prepared by dissolving 248 mM sucrose, 16.7 mM dextrose, 250 μM MgCl₂, and 100 μM CaCl₂ in deionised water, with osmolarity determined via the Osmomat 3000 (Gonotec, US) and adjusted to 290–299 mOsm. Phosphate buffered saline was added to adjust conductivity, measured with a Jenway 470 conductivity meter [37,38]. Cells were centrifuged at 125 G, and resuspended in fresh DEP medium, at two conductivities (43 mS/m and 103 mS/m), with final cell concentrations of 1 x 10^6^ cells mL^-1^ (± 15%).

HeLa and HCerEpiCs cells were analysed using a 3DEP cytometer (DEParator, UK) [20], where approximately 5 µL of cell suspension was loaded into each of the 20 wells of the DEP chip, covered with a glass slip, and inserted into the reader. Measurements were conducted across a range from 10 kHz to 45 MHz at 10V peak-to-peak for 30 seconds. DEP spectra from a minimum of three technical repeats were exported for analysis. ζ-potential measurements were undertaken using a Zetasizer Nano ZS90 (Malvern Panalytical, UK), with 800 µL of cell suspension in DEP medium loaded into disposable cuvettes. Several biological repeats were taken for each experimental group: untreated HCerEpiC (n = 4), 10 mM TEA treated HCerEpiC (n = 3), untreated HeLa (n = 9) and 10 mM TEA treated HeLa (n = 4). For each biological repeat, a minimum of three technical repeats were performed.

### 2.4 Electrical characterisation of cells

The HeLa and HCerEpiC populations were counted using a haemocytometer and the average cell radii of each cell subpopulation were measured using Image J software (NIH, Bethesda, Maryland). Technical repeats were averaged to provide a mean DEP spectrum for each study group. This spectrum was then fitted with a single-shell Clausius-Mossotti model [20,39,40] , a standard dielectric-electrophysiological model that relates the frequency-dependent DEP behaviour to the relationship between passive electrophysiological parameters. The best-fit parameter set yielded mean values of effective membrane capacitance per unit area (C_eff_), effective membrane conductance per unit area (G_eff_), and cytoplasmic conductivity (σ_Cyto_), plus the mean cell radius which was measured as described above. *V*_*m*_ was estimated using the σ_Cyto_ gradient as a function of medium conductivity [37].

### 2.5 Statistical analysis

Statistical analysis was undertaken using GraphPad Prism 9 software (Boston, USA). Data were presented as mean ± SEM. Dielectric parameters C_eff_, G_eff_, and σ_Cyto_, along with ζ-potential and modelled *V*_*m*_ values, were compared between HeLa and HCerEpiC groups (untreated versus 10 mM TEA treated). Linear regression models were used to determine trends between continuous data sets, where a student t-test was undertaken to determine the statistical significance of the slope. A one-way ANOVA test was undertaken to determine any significant differences in the electrophysiological parameters between all groups. Tukey’s Honest Significant Difference (HSD) post hoc test to analyse pairwise differences. T-tests was performed to determine statistical significance between a specific treatment group and the untreated control group. Statistical significance was observed when the P-value was ≤ 0.05.

## 3. Results and discussion

### 3.1 Membrane properties and cell radius

**Fig 2:**
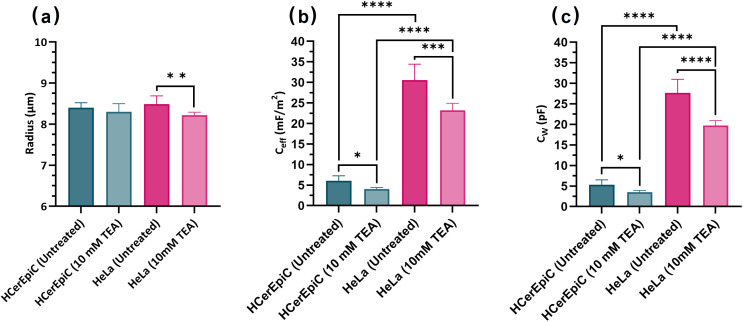
Cell radius (a), effective membrane capacitance (b), and whole-cell capacitance (c) of HeLa cervical carcinoma cells (pink) and primary HCerEpiCs (green) at 43 mS/m medium conductivity, either untreated or treated with 10 mM TEA. T-tests were undertaken to determine statistically significant differences between cell types and treatment groups. Sample numbers: untreated HCerEpiC (n = 4), 10 mM TEA treated HCerEpiC (n = 3), untreated HeLa (n = 9) and 10 mM TEA treated HeLa (n = 4). Error bars represent standard deviation.

As shown in Fig 2a, the cell radii of HeLa cells and HCerEpiCs were not significantly different, 8.5 ± 0.2 µm and 8.4 ± 0.1 µm respectively (*P > 0.05*). A statistically significant reduction in cell radius was only observed in HeLa cells treated with TEA (8.2 ± 0.1 µm, *P = 0.0056*), but not between untreated and treated HCerEpiCs (8.3 ± 0.2 µm, *P > 0.05*). The average C_eff_ (Fig 2b) of HeLa cells was 30.6 ± 3.9 mF/m^2^, which is significantly decreased to 23.2 ± 1.6 mF/m^2^ when treated with 10 mM TEA (*P = 0.0006*). The HCerEpiCs were measured at C_eff_ = 6.0 ± 1.2 mF/m^2^, significantly lower than the untreated HeLa cells (*P < 0.0001*). TEA treatment significantly reduced C_eff_ in HCerEpiCs to 4.0 ± 0.4 mF/m^2^ (*P = 0.041*). The one-way ANOVA showed there was a statistically significant difference in C_eff_ between study groups (F (3, 16) = 104.0, *P < 0.0001*). Tukey’s HSD identified significant differences in the C_eff_ between study groups, including: untreated HeLa and untreated HCerEpiCs (*P < 0.0001)*; TEA treated HeLa and HCerEpiCs (*P < 0.0001*) and untreated HeLa and TEA treated HeLa (*P < 0.005*).

The calculation of C_eff_ is based on a model that assumes membranes to be thin and flat; in fact, the microstructure of blebs and ruffles on the cell surface means there is more membrane per square micron of the assumed flat sphere, resulting in a higher *effective* membrane capacitance than the actual value with the “real” surface area. As a consequence, we can consider the elevation of C_eff_ beyond baseline as an indication of surface topography; indeed, Gascoyne et al presented the ratio of the two as the “folding factor” [41,42]. Here, we consider the value of C_eff_ as a marker in its own right, because of the usefulness in determining the whole-cell value C_w_.

The measured C_eff_ of HeLa cells aligned with measurements reported in the literature of around 17–39 mF/m^2^ [29,43,44]. We also calculated the whole-cell capacitance (C_w_; Fig 2c) of our samples by multiplying C_eff_ by the area of the hypothetical sphere used in the Clausius-Mossotti model, which has shown to accurately reproduce C_w_ for red blood cells [45]. This approach yielded a C_w_ for untreated HeLa cells of 27.6 ± 3.3 pF. This is significantly lower than published data using conventional patch clamp techniques, which ranges from 35–40 pF (*P < 0.0035*) [35,46]. However, this is in line with the observation of Hoettges et al. [20] that DEP-derived C_w_ measurements of cells with heterogeneous radii are typically 20–40% lower than values reported by patch clamp for the same cell type, whereas cell types with more homogeneous radii (such as red blood cells) showed a high degree of correlation between DEP and patch clamp measurements. The researchers posited that this is due to operator bias in the patch clamp technique, where operators (who are typically only able to measure a small number of cells per day) favour patching larger cells due to the difficulty of patching smaller cells, whereas DEP samples the entire population at scale.

The C_w_ of HeLa cells was approximately five times greater than normal HCerEpiCs, which was measured at 5.33 ± 1.15 pF respectively (*P < 0.0001*). This is consistent with prior established findings, where cells with a transformed or malignant phenotype typically exhibit elevated membrane capacitance to their normal counterparts [41,42,47,48]. Interestingly, the cell radii of untreated HeLa and HCerEpiCs were relatively similar (8.5 ± 0.20 µm versus 8.4 ± 0.12 µm), suggesting that the notable differences in C_eff_ and C_w_ between cell types are likely attributed to variations in membrane surface characteristics rather than differences in overall cell size.

Following TEA treatment, both HeLa and HCerEpiCs exhibited a significant decrease in C_eff_, approximately 24% and 33% respectively, suggesting alterations in cell morphology. A notable reduction in cell radius was observed specifically in the HeLa cells, which may suggest a more pronounced impact of K⁺ channel blockade on the malignant cell line compared to its normal counterpart. Interestingly, a reduction in C_eff_ after treatment is associated with a decrease in membrane folds, suggesting membrane stretching. Since this would be observable as an increase in measured radius – the converse of what was observed – it suggests that remodelling of the membrane, either physically or chemically, is responsible for the observed effect.

**Fig 3:**
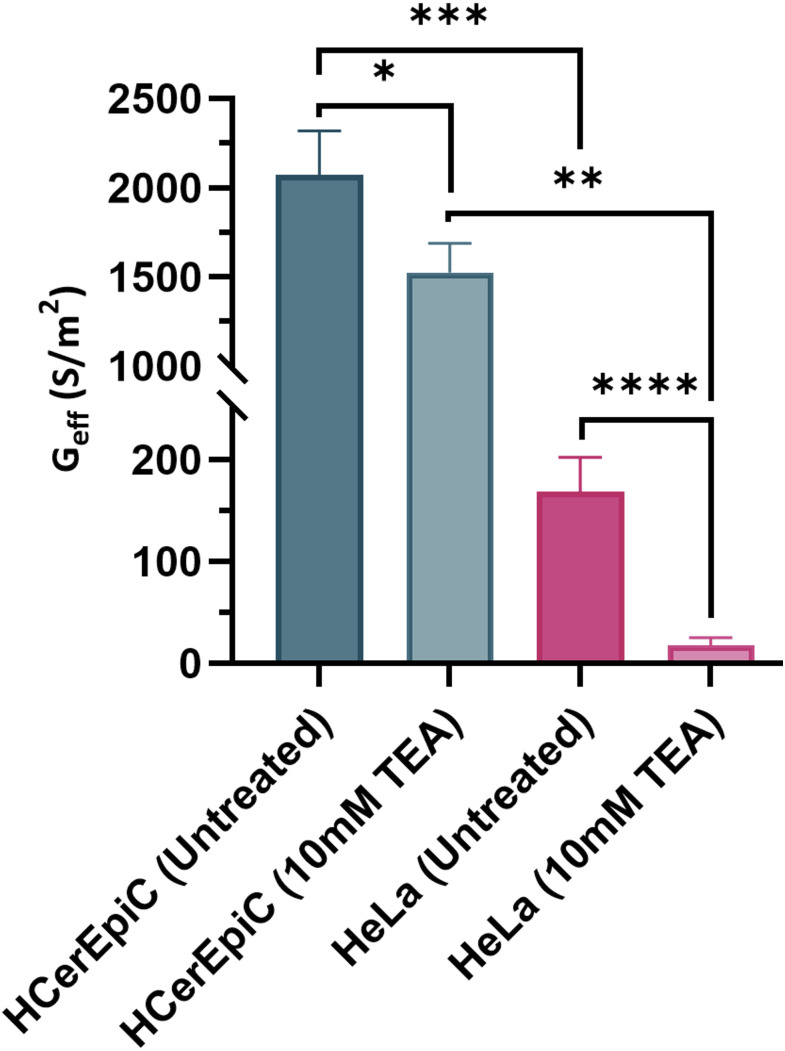
Effective membrane conductance (G_eff_) of HeLa cervical carcinoma cells (pink) and primary HCerEpiCs (green) at 43 mS/m medium conductivity, either untreated or treated with 10 mM TEA. T-tests were undertaken to determine statistically significant differences between cell types and treatment groups. Sample numbers: untreated HCerEpiC (n = 4), 10 mM TEA treated HCerEpiC (n = 3), untreated HeLa (n = 9) and 10 mM TEA treated HeLa (n = 4). Error bars represent standard deviation.

Fig 3 shows that G_eff_ of untreated HeLa cells was 168 ± 34 S/m, which declined by an approximate 89% when treated with 10 mM TEA (17.9 ± 7.1 S/m, *P < 0.0001*). In comparison, the G_eff_ of untreated HCerEpiCs was 2070 ± 248 S/m, approximately 12x greater than untreated HeLa cells (*P = 0.0005*). TEA treated HCerEpiCs had a reported 26.4% lower G_eff_ of 1520 ± 165 S/m (*P = 0.0005*). One-way ANOVA showed statistically significant difference in G_eff_ between study groups (F (3, 16) = 300.6, *P < 0.0001*). Tukey’s HSD identified many significant pairwise comparisons in G_eff_ values between groups, including: untreated HeLa and untreated HCerEpiCs (*P < 0.0001*); TEA treated HeLa and HCerEpiCs (P < 0.0001) and untreated HCerEpiCs and TEA treated HCerEpiCs *(P < 0.0005*). This suggests that untreated HeLa cells exhibited variations in ion channel activity and membrane composition, dissimilar to HCerEpiCs, resulting in significantly different G_eff_ values [41,49]; whilst the TEA treatment may have affected permeability of the cell membrane to ions, specifically K^+^, reducing the flow of ions across the membrane, hence reducing G_eff_ in both cell types. Measured changes in G_eff_ are commonly attributed to alterations in the ionic channels and membrane properties associated with cancer progression [41,49]. However, while it is well documented that cancer cells possess greater membrane capacitance compared to their normal counterparts, due to extensive morphological changes [42,47,50], changes in G_eff_ tends to be less consistent. For example, a DEP investigation by Liang et al. [51] on human oral cancer stem cells reported a linear increase in C_eff_ with tumorigenicity but no equivalent trend in G_eff_. Recent work [61] has posited that G_eff_ is more influenced by the cell membrane potential increasing surface charge by capacitive coupling, suggesting a more complex interplay of conductance, capacitance and potential. The G_eff_ of both cell types described here showed significantly reduction following treatment with 10 mM TEA, which likely indicates attenuated K^+^ transport across the membrane. This indicates that G_eff_ is a potential indicator for assessing relative changes within the same cell type, particularly in evaluating drug efficacy and cytotoxicity, as demonstrated in studies with ion channel blockers and anti-cancer drugs [29,45].

### 3.2 Cytoplasmic conductivity (σ_Cyto_)

**Fig 4:**
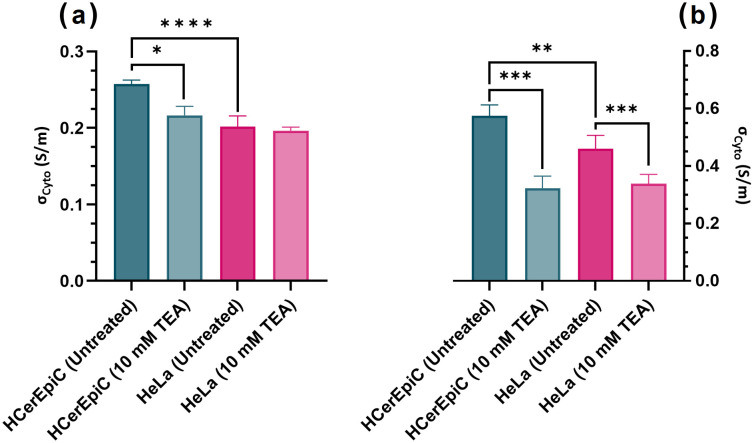
Cytoplasmic conductivity (σ_Cyto_) of HeLa cells (pink) and HCerEpiCs (green), either untreated or treated with 10 mM TEA, taken in medium conductivities of (a) 43 mS/m and (b) 103 mS/m. T-tests were undertaken to determine statistically significant differences between cell types and treatment groups. Sample numbers: untreated HCerEpiC (n = 4), 10 mM TEA treated HCerEpiC (n = 3), untreated HeLa (n = 9) and 10 mM TEA treated HeLa (n = 4). Error bars represent standard deviation.

As shown in Fig 4, there was a significant difference between the σ_Cyto_ of untreated HCerEpiCs (0.258 ± 0.005 S/m) compared to untreated HeLa cells (0.202 ± 0.014 S/m, *P < 0.0001*) and HCerEpiCs treated with 10 mM TEA (0.217 ± 0.012 S/m, *P = 0.0159*), at 43 mS/m medium conductivity. The one-way ANOVA identified statically significant difference between the study groups (F (3, 16) = 27.57, *P < 0.0001*). Tukey’s HSD reported significance between pairwise comparisons of untreated HCerEpiCs to untreated HeLa cells and untreated HCerEpiCs to TEA treated HCerEpiCs (*P < 0.005* for both comparisons). While the TEA treated HeLa cells reported a reduced σ_Cyto_ (0.196 ± 0.005 S/m), compared to untreated HeLa cells, the difference was non-significant at the medium conductivity of 43 mS/m (*P > 0.05*). However, when the medium conductivity was increased to 103 mS/m, the effects of TEA treatment appeared to have a more profound effect on σ_Cyto_; with a significant difference seen in σ_Cyto_ of untreated HeLa cells compared to TEA treated HeLa cells (0.46 ± 0.047 S/m versus 0.34 ± 0.033 S/m, *P = 0.0006*) and untreated HCerEpiCs and TEA treated HCerEpiCs (0.58 ± 0.038 versus 0.32 ± 0.042 S/m, *P = 0.001*). At the higher medium conductivity, the enhanced ion mobility in the extracellular environment may have increased the sensitivity of cells to ion channel blockers; conversely, this effect may be related to the correlation between *V*_*m*_ and σ_cyto_, with change of polarisation of the former being associated with a change in the latter. Similar trends were identified in the lower medium conductivity, where untreated HeLa cells exhibited a significantly lower σ_Cyto_ than untreated HCerEpiCs (*P = 0.002*); the one-way ANOVA showed a significant difference between study groups (F (3, 16) = 29.3, *P < 0.0001*). Tukey’s HSD identified several significant pairwise comparisons between untreated HeLa and HCerEpiCs, as well as each untreated cell type compared to its respective TEA treated group (*P < 0.005* for all).

### 3.3 ζ-potential and modelled *V*_*m*_

**Fig 5:**
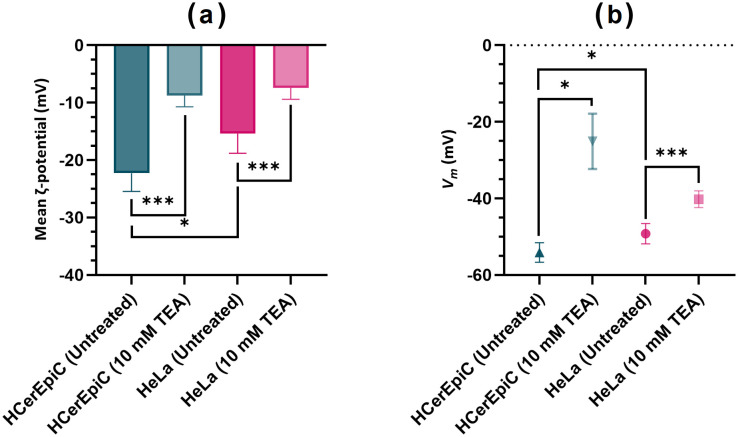
Mean ζ-potential (a) and estimated *V_m_* (b) of HeLa cervical carcinoma cells (pink) and primary HCerEpiCs (green), both untreated and treated with 10 mM TEA, taken at medium conductivity 43 mS/m. *V_m_* was estimated as described by Hughes et al [37]. T-tests were undertaken to determine statistically significant differences between cell types and treatment groups. Sample numbers: untreated HCerEpiC (n = 4), 10 mM TEA treated HCerEpiC (n = 3), untreated HeLa (n = 9) and 10 mM TEA treated HeLa (n = 4). Error bars represent standard deviation.

HeLa cells exhibited a significantly more depolarised ζ-potential (more than 30%) than untreated primary HCerEpiCs (*P = 0.0109*). There was also significant depolarisation in the ζ-potential of both cell types after treatment with 10 mM TEA, from −15.4 ± 3.5 mV to −7.4 ± 2.0 mV and −22.3 ± 3.15 to −8.8 mV in HeLa cells and HCerEpiCs respectively (Fig 5a), *P < 0.001* for both cell types. One-way ANOVA revealed significant differences in in the ζ-potential between study groups (F (3, 16) = 20.2, *P < 0.0001*). Tukey’s HSD identified several significant pairwise comparisons in ζ-potential, including untreated HeLa cells compared to TEA treated HeLa cells (*P < 0.005*); untreated HCerEpiCs and TEA treated HCerEpiCs (*P < 0.0005*) and significant differences between both untreated HeLa cells and HCerEpiCs (*P < 0.01*).

In order to estimate *V*_*m*_, the change in σ_cyto_ was determined as a function of change in medium conductivity, which was then processed using the equation of Hughes et al. [37]. There were noticeable differences in *V*_*m*_ between cell types (Fig 5b), with HeLa cells exhibiting a significant 9% greater depolarisation in *V*_*m*_ than primary HCerEpiCs at baseline (−49.2 ± 2.7 mV versus −54.1 ± 2.6 mV, *P = 0.02*). Furthermore, significant depolarisation was observed in both cell types when treated with 10 mM TEA, by 18.3% in treated HeLa cells (−40.2 ± 2.2 mV, *P = 0.*0003) and by 54% in treated HCerEpiCs (−25.1 ± 7.2 mV, *P = 0.014*). The one-way ANOVA also confirmed significant differences between cell types and treatment groups (F (3, 16) = 48.5, *P < 0.001*). Tukey’s HSD identified significant differences in *V*_*m*_ untreated HeLa cells and TEA treated HeLa cells (*P < 0.005*), as well as untreated HCerEpiCs and TEA treated HCerEpiCs (*P < 0.0001*). This suggests that *V*_*m*_ and ζ-potential of both HeLa and HCerEpiCs become more depolarized with TEA treatment, as blocking K⁺ channels prevent potassium efflux, causing the membrane potential to shift to a less negative value. This accumulation of positive charges also disrupts the surface charge balance, making the ζ-potential less negative. TEA thus affects both the internal electrical state and surface charge of cells.

*V*_*m*_ of untreated HeLa cells were highly consistent with patch clamp measurements in the literature (−49.2 ± 2.7 mV versus −51.0 to −48.2 mV) [52,53]. Furthermore, the HeLa cells were approximately 9% more depolarised than untreated normal primary HCerEpiCs. This is in line with the current understanding of membrane depolarisation as a signal to trigger cell cycle progression, inducing DNA synthesis and mitosis. Therefore, cancer cells typically exhibit a more depolarised *V*_*m*_, favouring proliferation, compared to their normal, non-transformed counterparts [31].

The ζ-potential of untreated HeLa cells was approximately 36.7% more depolarised than untreated HCerEpiCs, which is in line with reports by Bondar et al. [34] comparing several cancer cells (including HeLa) to normal epithelial cells. This is in line with observations that transformed/malignant phenotypes display a more depolarised ζ-potential [17]. Investigations by Hughes et al. [16] suggest there is a capacitive coupling effect across the cell membrane, where by modulating *V*_*m*_, cells can also change the surface charge on the lipid bilayer. This could mean that cells may use alteration of *V*_*m*_ to mechanistically change ζ-potential, to interact with its surroundings, such as in cell-to-cell interactions or other biomolecules. Untreated HeLa cells, with their more depolarized *V*_*m*_, likely experience a redistribution of ions near the cell surface, resulting in a more depolarized ζ-potential compared to HCerEpiCs. Although the exact reasons for cancer cells favouring a more depolarized ζ-potential are not fully understood, it may contribute to several factors, including enhanced migration and metastasis [54], and the attraction of nutrients, potentially promoting increased vascularization [55].

### 3.4 Diagnostic potential, and the effect of TEA

Whilst the results presented here are preliminary findings rather than a preclinical trial or pilot study, the presence of observable differences in the electrophysiological characteristics between HeLa cervical cancer cells and primary HCerEpiCs, produce distinct electrical “fingerprints” (Fig 1). These fingerprints, derived from DEP and ζ-potential measurements, demonstrate the potential of electrophysiological profiling as a rapid, label-free modality for characterizing and discriminating between healthy and malignant cells. HeLa cells exhibited an approximately five-times greater C_eff_ compared to HCerEpiCs (30.6 vs. 6.0 mF/m², P < 0.0001), consistent with the extensive membrane folding and increased surface area typical of cancer cells [47,48]. This elevated capacitance, a hallmark of malignant transformation, reflects morphological changes such as microvilli, ruffles, and protrusions that enhance cell proliferation and survival [50]. In contrast, G_eff_ was approximately twelve times lower in HeLa cells than in HCerEpiCs (168 vs. 2070 S/m, P = 0.0005). This is likely attributed to alterations in ionic channel composition, dysregulation of ion channels and transporters, *V*_*m*_ depolarisation, and altered membrane properties, collectively associated with cancer progression and metabolic adaptations.

**Fig 6:**
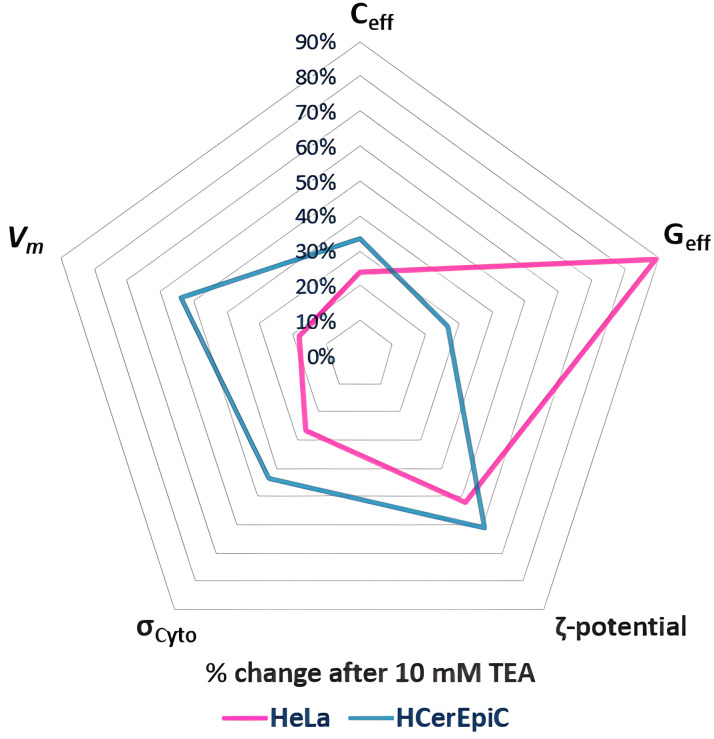
Radar plot comparing the effects of 10 mM TEA on HeLa cells and HCerEpiCs, presented as percentage change compared to untreated baseline of electrophysiological parameters: membrane capacitance (C_eff_), membrane conductance (G_eff_), cytoplasmic conductivity (σ_Cyto_), ζ-potential and estimated membrane potential (V_m_). The altered phenotype of malignant cells leads to distinct shifts in these parameters, differentiating them from their normal counterparts and demonstrating a novel approach to cellular profiling.

Treatment with the K⁺ channel blocker TEA further distinguished HeLa and HCerEpiCs via DEP and ζ-potential analysis, shown in Fig 6. TEA was observed to have significant effects on multiple aspects of cell electrophysiology of both cancerous and normal cells. It depolarised *V*_*m*_ of HCerEpiCs by 54%, but HeLa by only 18%, reflecting the lower cytosolic content of K+ in the latter case. This was reflected in the reduction in the *σ*_*cyto*_ values used to generate *V*_*m*_, which (at 103 mS/m) reduced by 44% and 24% respectively. Both cell types exhibited significant, broadly similar depolarisations in ζ-potential (of 61% in HCerEpiCs, 52% in HeLa), but substantially different reductions in G_eff_ (by 26% in HCerEpiCs but 89% in HeLa). The cells also showed a decrease in C_eff_, of 33% in HCerEpiCs and 24% in HeLa.

While both cell types exhibited *V*_*m*_ depolarisation following TEA treatment, the magnitude was far less in HeLa cells than HCerEpiCs (18% vs. 54% respectively), suggesting lower levels of cytosolic K^+^ in the cancer line, in accordance with expectations [30]. The depolarization also aligns with the likelihood of cell cycle arrest at the G2/M phase checkpoint [30]. The observed depolarization in the ζ-potential of both HeLa and HCerEpiCs after TEA treatment potentially indicates a reduction in the net negative charge on the cell surface. Whilst this may be due to a disruption in ionic homeostasis and cellular instability, it may also reflect a change in *V*_*m*_, which has been shown to be capacitively coupled to ζ-potential [16]. The difference in degree of change between ζ-potential and *V*_*m*_ in the two cell types may be attributable to the changes in C_eff_; since the two are believed to be capacitively coupled, the greater lower decrease in C_eff_ observed in HCerEpiCs as well as the lower initial baseline value may point to a different degree of coupling between the two parameters.

These differences in HeLa cell electrophysiology may also reflect adaptations to increase survivability compared to HCerEpiCs, which may contribute to their resistance to apoptosis and metabolic stress [49,56], and suggests that if DEP were used as a diagnostic, pretreatment by TEA may increase diagnostic accuracy. The results presented here suggest that DEP and ζ-potential analyses have potential to provide measurable parameters to distinguish cell types and monitor behavioural changes, positioning them as viable alternative or complementary profiling techniques to conventional diagnostic methods. A key advantage of these electrophysiological modalities is their cost-effectiveness compared to conventional molecular methods, enhancing accessibility in LMICs. A recent study (2023) examined the costs of multiple diagnostic modalities in Ghana [14], where the most commonly used diagnostic method was VIA, which cost around $3.50 per test, made up primarily of consumables costs. HPV tests and cytology were considerably more expensive at $23–24, where again the main associated cost was consumables. In Indonesia, a higher average cost of $18 per test was reported [15]. For comparison, a 3DEP chip in 2025 costs around $9, which could be reduced if manufactured at scale. Importantly, the diagnostic sensitivity of VIA is only 44%, compared to 97% for HPV detection and 73% for cytology. We have yet to determine diagnostic accuracy for cervical cancer in a clinical setting, but DEP-based methods for other epithelial carcinomas such as oral cancer [32] and bladder cancers [33] have shown sensitivity of over 80% and specificity of over 90%.

## 4. Conclusion

This investigation presents the first comprehensive measure assessment of the electrophysiological properties of HeLa cells and primary normal HCerEpiCs. Parameters such as C_eff_, ζ-potential, and modelled *V*_*m*_ were found to be significantly different between these cell types, reinforcing the potential as biomarkers to distinguish between healthy and diseased cells in a rapid, label-free, and cost-effective manner. The determination of *V*_*m*_ proved to be comparable to traditional patch clamp techniques, offering substantial improvements in throughput, ease of use, in a non-destructive manner. This approach introduces an exciting new and convenient modality to the electrophysiologist’s toolkit. There were several significant changes observed in the electrophysiological properties of both HeLa and HCerEpiCs when treated with TEA, notably the depolarization in *V*_*m*_ and ζ-potential, and a significant decrease in C_eff_. These changes, occurring at different magnitudes depending on cell type, suggest alterations in ion channel composition and function across the cell membrane, potentially indicating adaptations in HeLa cells that enhance their survivability.

## Supporting information

S1 File The experimental data set used in this paper. (PDF)
