# Supplementary material for: Electrophysiological fingerprints of healthy cervical epithelial and HeLa cells: Membrane potential, zeta potential and passive electrical properties
Source: PLoS One. 2025 Dec 17;20(12):e0337538. doi: 10.1371/journal.pone.0337538 (PMC12711043; doi:10.1371/journal.pone.0337538)
Supplement: S1 File — (PDF) [file pone.0337538.s001.pdf]

Mean Radius (μm)	G_{eff} 43 (S/m^2)	G_{eff} 103 (S/m^2)	R_{wc} 43 (MΩ)	R_{wc} 103 (MΩ)	C_{eff} 43 (mF/m^2)	C_{eff} 103 (mF/m^2)	C_{wc} 43 (pF)	C_{wc} 103 (pF)	Zeta 43 (mV)	σ_{Cyto} 43 (S/m)	σ_{Cyto} 103 (S/m)	V_m (mV)
**HeLa (untreated)**												
8.2365	171.43	214.29	5.47	6.84	24.032	32.886	20.5	32.886	-14.595	0.235	0.58	-55.6009
8.6345	228.57	185.71	5.75	4.67	26.562	35.416	24.9	35.416	-12.6	0.19	0.48	-50.7258
8.4985	185.71	142.86	7.71	5.93	30.357	26.562	27.6	26.562	-16.04	0.192	0.448	-49.2707
8.485	142.86	142.86	7.74	7.74	29.092	29.092	26.3	29.092	-19.867	0.19	0.438	-48.8826
8.82	142.86	257.14	3.98	7.16	30.357	31.621	29.7	31.621	-14.689	0.21	0.44	-47.7949
8.674	142.86	271.43	3.9	7.4	30.357	29.092	28.7	29.092	-10.53	0.2	0.438	-47.8408
8.2035	142.86	142.86	8.28	8.28	36.048	34.151	30.5	34.151	-19.658	0.2	0.438	-48.4047
8.445	214.29	142.86	7.81	5.21	32.886	29.092	29.5	29.092	-18.648	0.2	0.438	-47.8229
8.35	142.86	142.86	7.99	7.99	35.416	24.032	31	24.032	-11.73	0.2	0.435	-46.6105
**HeLa (10 mM TEA)**												
8.3015	14.286	42.857	26	80.8	21.503	17.708	18.62182	15.87946	-10.221	0.2	0.352	-40.9618
8.136	14.286	42.857	27.5	84.2	24.665	18.72	20.51695	15.8679	-5.499	0.19	0.352	-40.714
8.23	14.286	71.429	16.5	82.2	24.665	20.238	20.99378	17.16298	-6.684	0.2	0.36	-42.0978
8.218	28.571	14.286	78.5	41.2	22.135	8.854	18.78545	7.897492	-7.26	0.195	0.29	-37.0566
**HCerEpiC (Untreated)**												
8.4975	2285.7	2857.1	0.383694	0.482157	6.0713	5.8183	5.509014	5.307441	-18.895	0.25	0.6	-56.3242
8.4475	2285.7	7142.9	0.154853	0.487882	4.9329	4.427	4.423526	4.002358	-24.845	0.26	0.58	-54.0212
8.428	1857.1	5714.3	0.188884	0.603262	7.7156	9.6129	6.886977	8.906286	-25.09	0.26	0.52	-50.5894
8.22	1857.1	5571.4	0.196903	0.634178	5.31	7.4627	4.510703	6.802666	-20.34	0.26	0.6	-55.5792
**HCerEpiC (10 mM TEA)**												
8.52	1428.6	2714.3	0.433899	0.767362	4.3005	5.5654	3.922907	4.725523	-9.0725	0.23	0.37	-32.7756
8.14	1714.3	2857.1	0.420355	0.700575	4.17	8.4745	3.475451	7.05623	-10.51	0.21	0.31	-24.1282
8.24	1428.6	2857.1	0.410214	0.820399	3.54	6.1978	3.021787	5.288127	-6.622	0.21	0.29	-18.3934

Supplementary information.

The experimental data set used in this paper. Pink columns taken at 43 mS/m, blue at 103 mS/m. Green is independent of conductivity.
